# Towards predicting the lung fibrogenic activity of nanomaterials: experimental validation of an in vitro fibroblast proliferation assay

**DOI:** 10.1186/1743-8977-10-52

**Published:** 2013-10-10

**Authors:** Giulia Vietti, Saloua Ibouraadaten, Mihaly Palmai-Pallag, Yousof Yakoub, Christian Bailly, Ivana Fenoglio, Etienne Marbaix, Dominique Lison, Sybille van den Brule

**Affiliations:** 1 Louvain centre for Toxicology and Applied Pharmacology, Université catholique de Louvain, Avenue E. Mounier, 52 - bte B1.52.12, 1200 Brussels, Belgium; 2 Bio and soft matter, Institute of Condensed Matter and Nanosciences, Université catholique de Louvain, Croix du Sud 1 - bte L7.04.02, 1348 Louvain-la-Neuve, Belgium; 3 Interdepartmental Center “G. Scansetti” for Studies on Asbestos and other Toxic Particulates, Università degli Studi di Torino, Via P. Giuria 7, Torino, 10125, Italy; 4 De Duve Institute, Université catholique de Louvain, Avenue Hippocrate 75 - bte B1.75.02, 1200 Brussels, Belgium

**Keywords:** Carbon nanotubes, CNT, Lung fibrosis, Asbestos, Crocidolite, Dispersion, MLg, WST-1

## Abstract

**Background:**

Carbon nanotubes (CNT) can induce lung inflammation and fibrosis in rodents. Several studies have identified the capacity of CNT to stimulate the proliferation of fibroblasts. We developed and validated experimentally here a simple and rapid in vitro assay to evaluate the capacity of a nanomaterial to exert a direct pro-fibrotic effect on fibroblasts.

**Methods:**

The activity of several multi-wall (MW)CNT samples (NM400, the crushed form of NM400 named NM400c, NM402 and MWCNTg 2400) and asbestos (crocidolite) was investigated in vitro and in vivo. The proliferative response to MWCNT was assessed on mouse primary lung fibroblasts, human fetal lung fibroblasts (HFL-1), mouse embryonic fibroblasts (BALB-3T3) and mouse lung fibroblasts (MLg) by using different assays (cell counting, WST-1 assay and propidium iodide PI staining) and dispersion media (fetal bovine serum, FBS and bovine serum albumin, BSA). C57BL/6 mice were pharyngeally aspirated with the same materials and lung fibrosis was assessed after 2 months by histopathology, quantification of total collagen lung content and pro-fibrotic cytokines in broncho-alveolar lavage fluid (BALF).

**Results:**

MWCNT (NM400 and NM402) directly stimulated fibroblast proliferation in vitro in a dose-dependent manner and induced lung fibrosis in vivo. NM400 stimulated the proliferation of all tested fibroblast types, independently of FBS- or BSA- dispersion. Results obtained by WST1 cell activity were confirmed with cell counting and cell cycle (PI staining) assays. Crocidolite also stimulated fibroblast proliferation and induced pulmonary fibrosis, although to a lesser extent than NM400 and NM402. In contrast, shorter CNT (NM400c and MWCNTg 2400) did not induce any fibroblast proliferation or collagen accumulation in vivo, supporting the idea that CNT structure is an important parameter for inducing lung fibrosis.

**Conclusions:**

In this study, an optimized proliferation assay using BSA as a dispersant, MLg cells as targets and an adaptation of WST-1 as readout was developed. The activity of MWCNT in this test strongly reflects their fibrotic activity in vivo, supporting the predictive value of this in vitro assay in terms of lung fibrosis potential.

## Background

Serious concerns have been raised regarding the potential adverse effects of nanomaterials (NM) on the health of exposed workers, users or consumers, as well as on the environment [1]. Among the variety of already existing NM, carbon nanotubes (CNT) are particularly scrutinized because of their structural resemblance to asbestos, suggesting that they could induce similar harmful effects, including inflammatory and fibrotic lung reactions, pleural mesothelioma and lung cancer [2]. Furthermore, pristine CNT are generally hydrophobic, essentially insoluble in water and, when long enough, expected to be biopersistent. These features contribute to reinforce the concerns about their pathogenic activity [3].

Most experimental studies conducted with CNT have focused on respiratory effects because inhalation represents the most worrying route of exposure to this material. Early studies indicated that they were able to induce inflammatory reactions, granulomas, fibrosis, and biochemical changes in rodent lungs when administered intratracheally or intrapharyngeally [4-6]. In these studies CNT were shown to be more toxic than quartz, well known for its pulmonary toxicity. Results from inhalation studies revealed the capacity of CNT to induce lung fibrosis [7,8] or not [9-11].

Recent data indicated that CNT can stimulate lung fibroblast proliferation in vitro. Wang et al. [12] showed that lung fibroblasts exposed in vitro to single-wall (SW)CNT displayed increased proliferation and collagen production in the absence of cell damage. They also observed that SWCNT stimulated the proliferation of lung epithelial cells. Non-dispersed SWCNT were not active in these assays, pointing to the importance of isolated CNT for these effects [13]. Similar results were obtained with multi-wall (MW)CNT [14-16].

This in vitro activity of CNT is likely relevant to understand their capacity to induce lung fibrosis in vivo because fibroblast proliferation is an essential component of the fibrotic process, together with differentiation in myofibroblasts and excessive production of collagen, leading to the exaggerated deposition of extracellular matrix [17-19]. Though inhaled NM first encounter macrophages and epithelial cells in alveoli, a direct interaction of CNT with fibroblasts is very likely in vivo since it has been shown that they can rapidly reach the alveolar interstitial space and co-localize with inflammatory and fibrotic granulomas containing collagen fibers [4,5,20].

The capacity of CNT (or other NM) to stimulate fibroblast proliferation may, therefore, offer an opportunity to develop an in vitro assay able to predict a fibrotic activity in vivo. Such an in vitro assay would contribute to the efforts for reducing the number of toxicological testings on vertebrates to ensure the protection of human health, including in the context of the European REACH regulations.

In this manuscript, we report on the development of a simple, effective, fast and standardized in vitro assay to determine whether a NM can exert a direct proliferative effect on fibroblasts. The consistency of the proliferative response was confirmed with different endpoints, cell types and dispersion conditions. The association between the proliferative activity of CNT on fibroblasts in vitro and their ability to induce pulmonary fibrotic responses in vivo was established by correlating the respective activities of different samples.

## Results

### Physicochemical characterization of MWCNT

**Figure 1:**
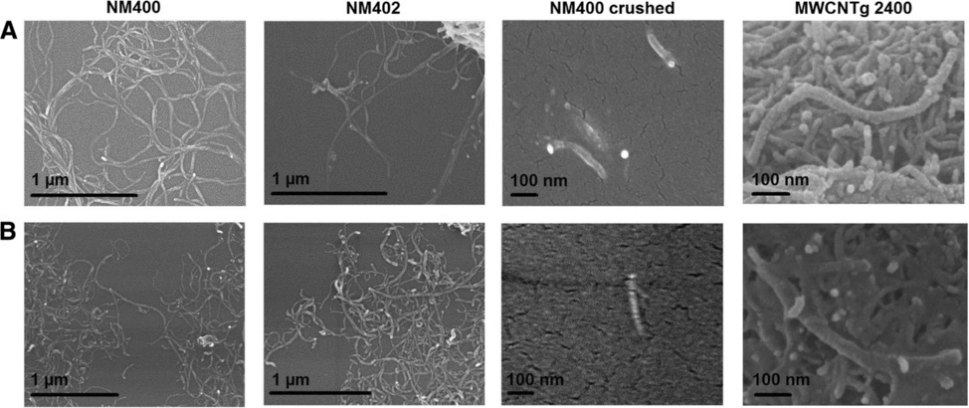
**Scanning Electron Microscopy images of different MWCNT.** NM400, NM402, NM400c and MWCNTg 2400 images were taken **(A)** before and **(B)** after dispersion by sonication with a scanning electron microscope.

The morphology of the different MWCNT samples is shown in Figure 1 and their characteristics are reported in Table 1. NM400 and NM402 are similar in length but slightly differ by their diameter and impurity content. MWCNTg 2400 were shorter than NM400 and NM402 and had lower defect and impurity levels. The individual length of crushed NM400 (NM400c) was significantly reduced compared to the pristine NM400, without modifying other characteristics. CNT diameters and lengths were not modified after the dispersion by sonication (Figure 1B).

**Table 1:** Physicochemical characterization of MWCNT

	**NM400**	**NM402**	**NM400 crushed**	**MWCNTg 2400**^**b**^
Diameter (nm)	5 – 35^a^	6 - 20^a^	18 - 35	20 - 50
Length (μm)	0.7 - 3^a^	0.7 - 4^a^	0.14 - 0.5	0.7
Impurities	5.38	3.16	5.22	0.37
% Al	0.24	3.00 x 10^-4^	0.24	0.15 x 10^-3^
% Co	0.58	2.39	0.50	0.10 x 10^-4^
% Fe				
Extent of defects (I_D_/I_G_)	1.20	1.12	1.20	0.58

Refer to [42]^a^ and [24,25]^b^.

### MWCNT stimulate fibroblast proliferation in vitro and induce lung fibrosis

**Figure 2:**
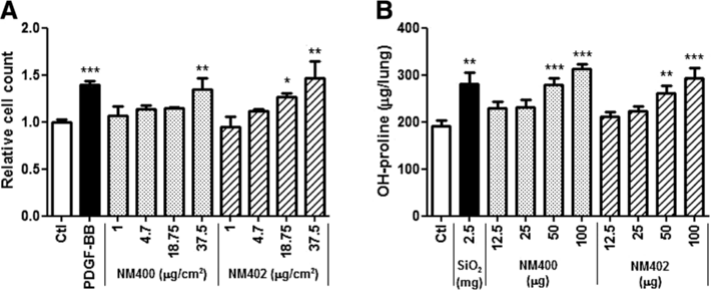
**MWCNT stimulate fibroblast proliferation in vitro and induce lung fibrosis in a dose-dependent manner. (A)** In vitro effect of MWCNT on primary murine lung fibroblast proliferation assessed by cell counting. Fibroblasts cultured from C57BL/6 mouse lungs were treated with different doses (μg/cm^2^ culture well) of MWCNT (NM400 and NM402, final FBS concentration 0.2%) for 24 h. PDGF-BB (50 ng/ml) was used as a positive control. Results are expressed relative to non-treated cell number (Ctl). * p < 0.05, ** p < 0.01, *** p < 0.001 vs non-treated cells (n = 3, t-test or Dunnet multiple comparison test as appropriate). **(B)** In vivo lung fibrotic response to MWCNT (hydroxyproline lung content) assessed 2 months after pharyngeal administration in mice with different doses of MWCNT (NM400 and NM402), or Min-U-Sil (SiO_2_) as positive control. * p < 0.05, ** p < 0.01, *** p < 0.001 vs non-treated cells (n = 5-7, t-test or Dunnet multiple comparison test as appropriate).

The (pro-)fibrotic activity of NM400 and NM402 was assessed in vitro on fibroblasts and in vivo in mouse lungs. Primary lung fibroblasts were exposed to NM400 and NM402 pre-dispersed in 2% fetal bovine serum (FBS - 0.2% final FBS concentration during cell exposure), or to human platelet-derived growth factor (PDGF)-BB as a fibrosis-relevant growth factor. Cell proliferation was assessed by counting the number of living cells after 24 h exposure to doses ranging from 1 to 37.5 μg/cm^2^. Both CNT samples significantly increased cell number in a dose-dependent manner and the highest concentration induced an effect similar to PDGF (Figure 2A). In parallel, the pulmonary fibrotic response to the same CNT was assessed. Min-U-Sil (SiO_2_), a reference micrometric crystalline silica particle, was used as positive control. Fibrosis was assessed by quantifying hydroxyproline lung content as a marker of total collagen accumulation, 2 months after instillation. NM400 and NM402 induced a dose-dependent collagen accumulation and, at the highest doses (50–100 μg/mouse), similar in extent to 2.5 mg Min-U-Sil (Figure 2B). Thus, the tested MWCNT showed a direct effect on lung fibroblasts which might reflect their fibrotic activity in vivo. We next optimized the experimental conditions for the in vitro proliferation assay.

### Influence of the dispersion medium on the in vitro effect of MWCNT on fibroblast proliferation

**Figure 3:**
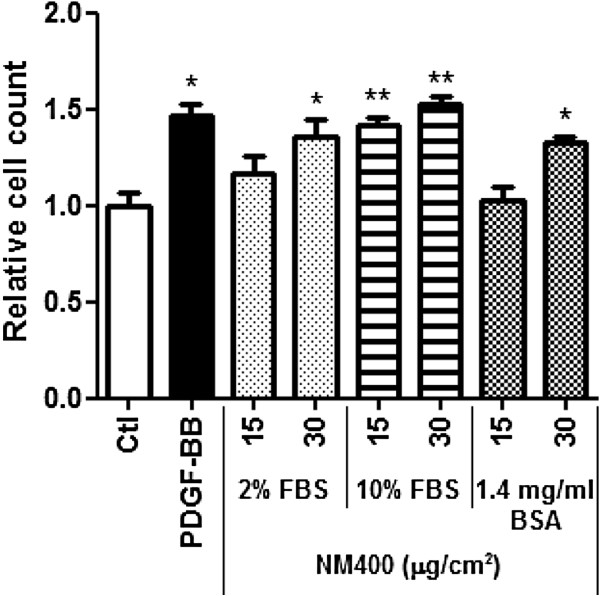
**Influence of dispersion medium on the in vitro effect of MWCNT on fibroblast proliferation assessed by cell counting.** Primary murine lung fibroblasts were treated for 24 h with different doses of NM400 dispersed in 2 or 10% FBS or 1.4 mg/ml BSA (final concentrations 0.2% FBS and 0.14 mg/ml BSA, respectively). PDGF-BB (50 ng/ml) was used as a positive control. Results are expressed relative to non-treated cell number (Ctl). * p < 0.05, ** p < 0.01 vs non-treated cells (n = 3, t-test or Dunnet multiple comparison test as appropriate).

Since NM400 and NM402 similarly increased fibroblast proliferation and induced similar lung fibrosis, subsequent in vitro experiments were mainly performed with NM400. To exclude any FBS-specific effect, e.g. linked to a growth factor activity present in serum and possibly adsorbed on MWCNT, several other dispersion media were compared. NM400 was dispersed in FBS 2% or 10%, bovine serum albumin (BSA) 1.4 mg/ml, or Survanta 150 μg/ml (a natural bovine lung extract used to mimic lung surfactant). The selected BSA concentration is based on the total protein concentration present in FBS 2%. Figure 3 shows that NM400 induced fibroblast proliferation with all dispersion media containing FBS or BSA. The effect was, however, amplified with increasing serum concentration in the dispersion medium. The proliferation of fibroblasts (BALB-3T3) was also increased by CNT dispersed in Survanta (data not shown). These results supported the selection of 1.4 mg BSA/ml to disperse test materials for subsequent experiments, since it excludes that active serum components might confound the proliferation effect.

### Selection of a fibroblast cell line

**Figure 4:**
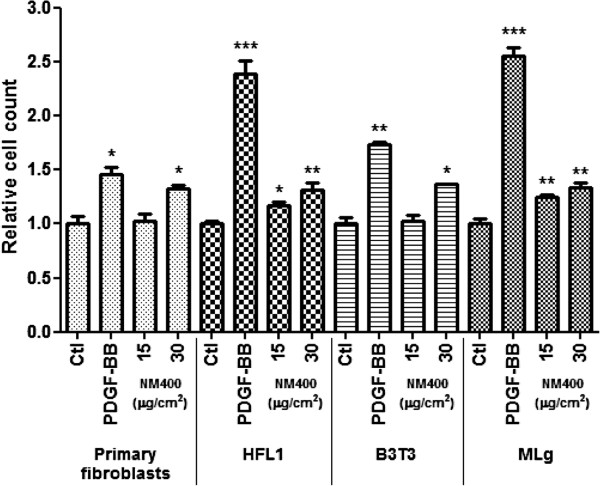
**In vitro effect of MWCNT on proliferation of several fibroblast types assessed by cell counting.** Primary murine lung fibroblasts and human and mouse fibroblast cell lines (HFL-1, BALB/3T3 and MLg) were treated for 24 h with different doses of NM400 dispersed in 1.4 mg BSA /ml (final concentration 0.14 mg BSA /ml). PDGF-BB (50 ng/ml) was used as a positive control. Results are expressed relative to non-treated cell number (Ctl). * p < 0.05, ** p < 0.01, *** p < 0.001 vs non-treated cells (n = 3, t-test or Dunnet multiple comparison test as appropriate).

To examine the response of different fibroblast cell types, the effect of NM400 on human fetal lung fibroblasts (HFL-1), mouse embryonic fibroblasts (BALB-3T3) and mouse lung fibroblasts (MLg) was evaluated. At the concentrations tested, NM400 had a similar and significant stimulating effect on the proliferation of all fibroblasts (Figure 4). MLg were chosen for subsequent experiments since the propagation of this cell in culture is more convenient than primary cells and furthermore, the in vitro results obtained with these cells can be directly compared to in vivo experiments performed in mice.

### Selection and validation of an optimal proliferation assay

**Figure 5:**
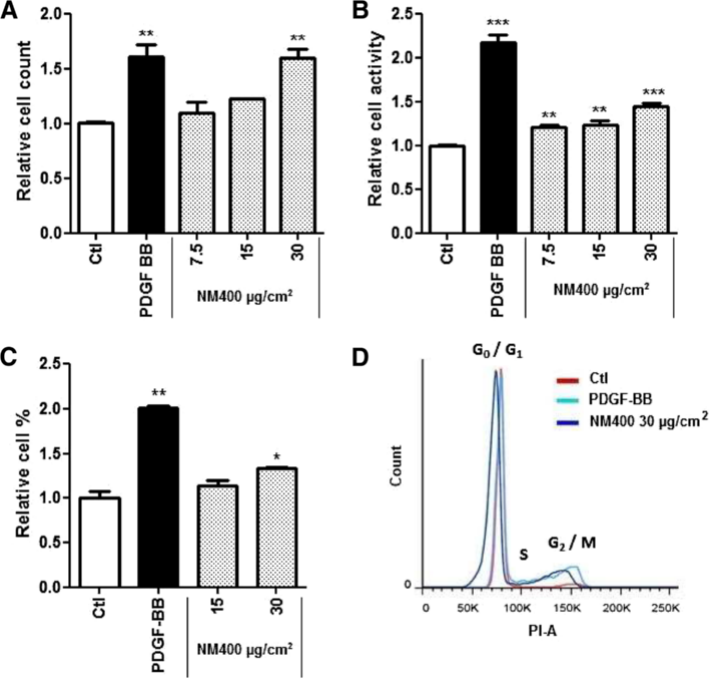
**In vitro effect of MWCNT on MLg fibroblast proliferation assessed by different assays.** Fibroblasts were treated 24 h with different doses of NM400 dispersed in 1.4 mg BSA /ml (final concentration 0.14 mg BSA /ml). **(A)** Cell counting, **(B)** WST-1 assay and **(C, D)** propidium iodide staining **(C)** % cells in S and G2/M phases and **(D)** histogram plot event counts vs PI were used for in vitro assays. PDGF-BB (30 ng/ml) was used as a positive control. Results are expressed relative to cell number, WST1 activity or % cells in S/G2/M in non-treated cells (Ctl). * p < 0.05, ** p < 0.01, *** p < 0.001 vs non-treated cells (n = 3-4, t-test or Dunnet multiple comparison test as appropriate).

Accumulating evidence show that CNT can interact with conventional in vitro assays leading to potentially biased results [21-23]. This may be avoided by evaluating the in vitro proliferative activity by counting cells, as this readout appears as the least susceptible to CNT interference. However, since manual cell counting is time consuming and not amenable to large throughput, we explored the possibility to use other assays by using cell counting as a reference method. The relevance of other in vitro endpoints, including water-soluble tetrazolium salts (WST)-1, Alamar Blue, CyQuant, [^3^H]-thymidine incorporation and propidium iodide (PI) staining was further assessed. Alamar blue assay yielded unstable results, and MWCNT interfered with most other assays. We observed that MWCNT can interfere with different aspects of the experimental procedures, such as the assay absorbance wavelength (WST-1), DNA nucleosides (thymidine) and DNA intercalating agents (CyQuant) (data not shown). However, we were able to fend off most potential interferences of MWCNT with the WST-1 assay by applying the procedure described in the Methods section. Figure 5B shows that WST-1 activity increased dose-dependently in response to MWCNT, similarly to cell number (Figure 5A), suggesting that this adapted procedure can be used instead of cell counting, particularly if high throughput is sought.

At the same time, increased cell number after exposure to MWCNT was confirmed to be, at least in part, due to enhanced proliferation by using PI staining that evaluates the cell DNA content. The ratio of fibroblasts in S (DNA synthesis) and G_2_/M (Gap2 and mitosis) phases 24 h after exposure was calculated from the histogram (event counts vs. PI fluorescence) and was significantly increased after treatment with PDGF and NM400 (Figure 5C and D). Figure 5D shows a representative histogram that illustrates FACS data for each condition. Thus, data obtained by cell counting were confirmed by two other readouts based on completely different principles, and proliferation can be conveniently assessed by the adapted WST-1 assay.

### In vitro comparison of different CNT samples

**Figure 6:**
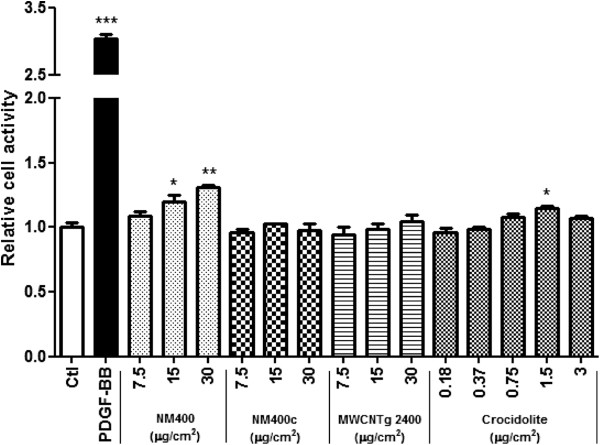
**In vitro effect of several MWCNT and crocidolite on MLg fibroblast proliferation.** Fibroblasts were treated for 24 h with different doses of MWCNT (NM400, NM400c, MWCNTg 2400) and crocidolite dispersed in 1.4 mg BSA /ml (final concentration 0.14 mg BSA /ml). WST-1 assay were used for in vitro assays. PDGF-BB (30 ng/ml) was used as a positive control. Results are expressed relative to WST1 activity in non-treated cells (Ctl). * p < 0.05, ** p < 0.01, *** p < 0.001 vs non-treated cells (n = 3-4, t-test or Dunnet multiple comparisons test as appropriate).

To assess the effect of CNT length on fibroblasts proliferation, we disrupted the “high-aspect ratio” structure of NM400 by grinding (crushed NM400, NM400c). Additionally, we selected MWCNTg 2400 [24,25], characterized by a length (0.7 μm) intermediate between NM400 and NM400c. Crocidolite was included because of its known (pro-)fibrotic activities in vitro and in vivo and of its fiber-like structure (in the micrometer length range). Figure 6 shows that intact NM400 and crocidolite, but not NM400c and MWCNTg 2400, stimulated fibroblast proliferation as shown by the WST-1 assay. NM400 had a stronger proliferative activity on fibroblast cells than crocidolite.

### The in vitro effect on fibroblast proliferation appears predictive of the in vivo lung fibrotic response to MWCNT

**Figure 7:**
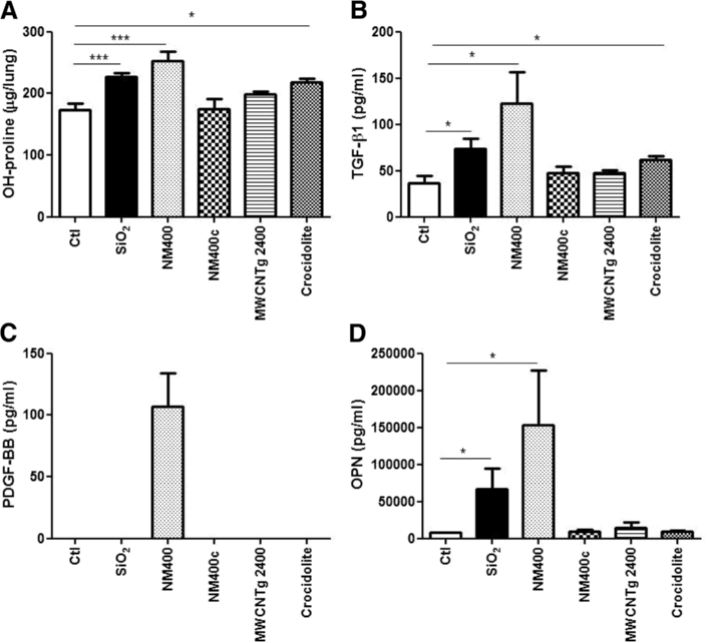
**In vivo lung fibrotic response to several MWCNT and crocidolite. (A)** Hydroxyproline lung content and cytokine levels in BALF, **(B)** total TGF-β1, **(C)** PDGF-BB and **(D)** OPN (quantified by ELISA) were measured 2 months after pharyngeal aspiration of MWCNT (NM400, NM400c, MWCNTg 2400) and crocidolite in C57BL/6 mice. Min-U-Sil (SiO_2_) was used as positive control. * p < 0.05, ** p < 0.01, *** p < 0.001 vs non-treated mice (n = 5-8, t-test).

**Figure 8:**
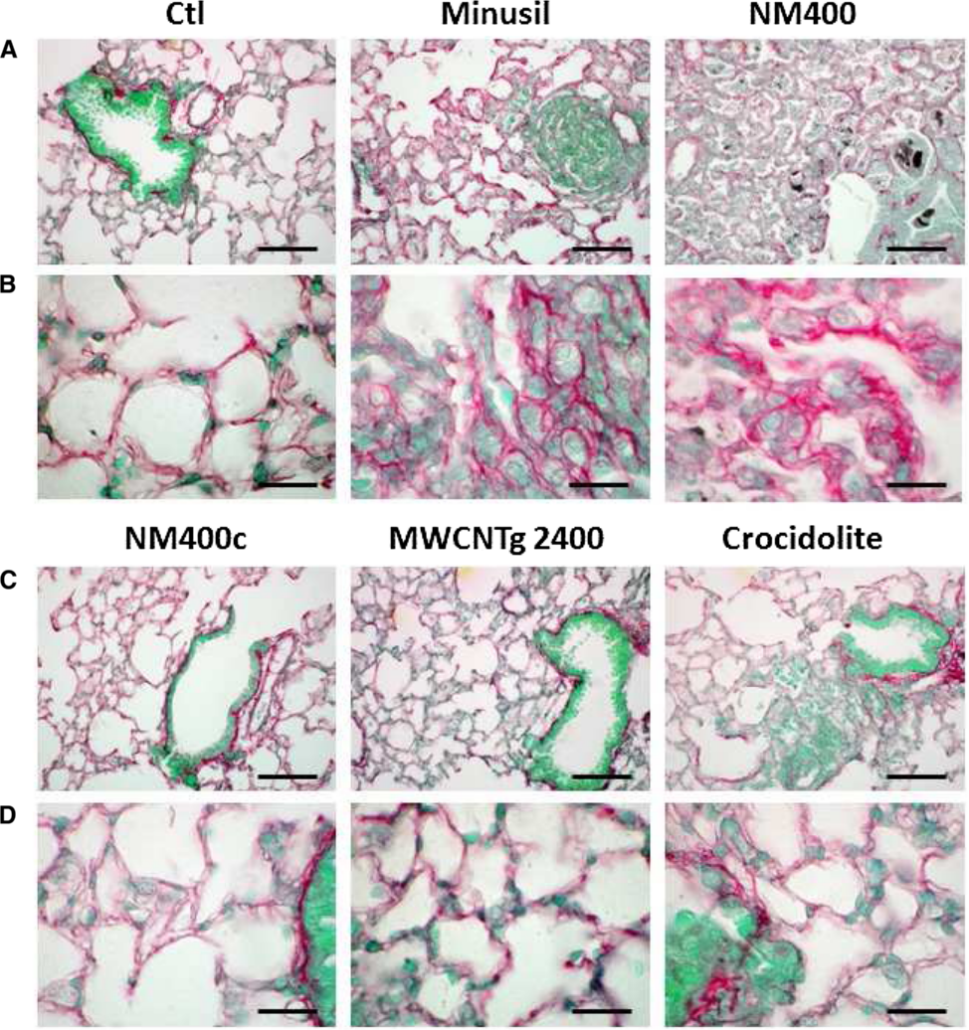
**In vivo lung fibrotic response to several MWCNT and crocidolite assessed by histology.** Sirius red lung sections were analyzed 2 months after pharyngeal aspiration of MWCNT (100 μg - NM400, NM400c and MWCNTg 2400) and crocidolite (100 μg) in C57BL/6 mice. Min-U-Sil (SiO_2_) was used as positive control. Bars correspond to 100 μm **(A, C)** or 20 μm **(B, D)**.

In parallel, C57BL/6 mice were aspirated with 100 μg of each of these CNT samples; silica (Min-U-Sil) and asbestos (crocidolite) were used as positive controls. Two months after treatment, we observed a significant increase of collagen lung content with the intact NM400, silica and crocidolite, but not with NM400c or MWCNTg 2400 (Figure 7A). The histopathological study, evaluated by Sirius red staining, confirmed the biochemical findings, revealing the accumulation and a greater thickness of the collagen fibers only in the lung of mice instilled with NM400 and crocidolite (Figure 8) tough NM400 led to a stronger fibrotic response than crocidolite. Furthermore, the levels of total transforming growth factor (TGF)-β1, PDGF-BB and osteopontin (OPN), all key mediators of the lung fibrotic process, were significantly increased in the bronchoalveolar lavage fluids (BALF) of mice exposed to intact NM400 and to a greater extent than to silica and crocidolite (Figure 7B to D). NM402 also enhanced these cytokines in a dose-dependent manner (data not shown).

Altogether, these results indicate that in vitro fibroblast proliferation and lung fibrosis appear to be mainly induced by intact CNT and to a lower extent by asbestos. Furthermore, they suggest a predictive value of the in vitro proliferative activity in terms of lung fibrosis potential.

## Discussion

Several in vivo studies have shown that CNT can induce a fibrotic reaction in the lung (for a review see [2,26,27]). A number of investigators have indicated that the mechanism of CNT-induced pulmonary fibrosis is indirect and mediated via macrophages and/or epithelial cells. Cesta et al. [28] demonstrated that pre-existing respiratory inflammation exacerbates the lung fibrotic response to CNT by increasing PDGF release by macrophages and the PDGF-receptor in fibroblasts. More recently, Li et al. [29] showed that macrophages and epithelial cells exposed to CNT can release interleukin (IL)-1β, TGF-β and PDGF, three key mediators in the development of pulmonary fibrosis. Wang et al. [30] also reported that long MWCNT can activate macrophages and increase their production of TGF-β.

Other investigators have shown the ability of CNT to reach the alveolar interstitium and to directly enter in contact with fibroblasts [5,20]. It is, therefore, relevant to examine whether CNT can act directly on fibroblasts, in addition to the indirect inflammatory pathway. CNT have already been reported to stimulate the in vitro proliferation of lung fibroblasts [12,16], epithelial cells [13] and osteoblasts [31,32]. In the present study, we confirmed the ability of MWCNT to stimulate fibroblast proliferation and we optimized an in vitro assay to assess this activity. The response to MWCNT in this assay appears predictive of their capacity to induce pulmonary fibrosis in vivo, which further supports the biological relevance of this assay.

**Figure 9:**
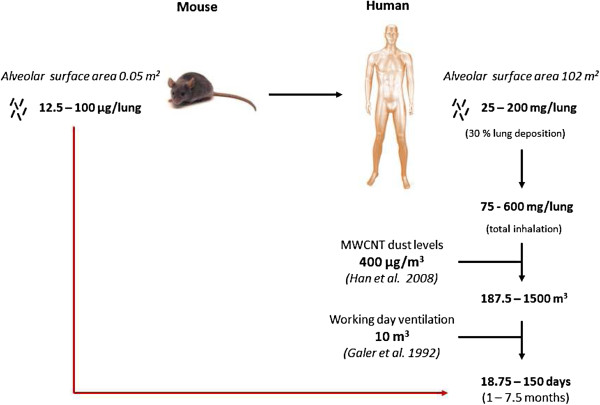
Correlation and relevance of in vivo mouse doses to human MWCNT exposure.

**Figure 10:**
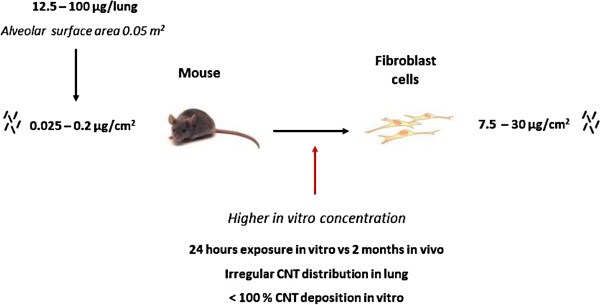
Correlation and relevance of in vivo mouse exposure to in vitro fibroblast study.

Whether doses applied in the present study are relevant for human exposure scenarios can be evaluated by the approach suggested by Porter et al. [33] (Figure 9). Mice were exposed to 12.5-100 μg/mouse (alveolar surface area 0.05 m^2^), which is equal to 25-200 mg/lung in humans (alveolar surface area 102 m^2^). Considering a deposition fraction of 30% [34], a peak MWCNT airborne level of 400 μg/m^3^ measured in a research laboratory [35], and a minute ventilation of 20 l/min [36], a human- equivalent dose would be achieved within 1 to 7.5 months of exposure in a contaminated work place. Assuming a 10-fold lower exposure of 40 μg/m^3^, the experimental dose could be reached after 10 months to 6.2 years of human inhalation exposure. Extrapolation of in vitro concentrations to in vivo dosimetry can be approximated by normalizing the surface area exposed to MWCNT in the tissue culture dish and in the murine airways [16]. Here, the in vivo doses correspond to 0.025-0.2 μg MWCNT/cm^2^ culture well, which is less than the in vitro doses used (7.5 - 30 μg/cm^2^). However, three important aspects should be taken into account: (i) in vitro, cells are exposed for only 24 h, while in vivo experiments were performed over 2 months, (ii) in vivo, CNT are not distributed uniformly in the lung and inhaled particles can be found concentrated in some so-called hot spots in the lung [37] and (iii) it should also be considered that CNT do not entirely sediment onto cells in vitro. These aspects can, therefore, contribute to justify the doses used in vitro to explore the possible mechanisms of CNT toxicity in the lung (see Figure 10).

The doses used in the present study both in vitro (7.5 - 30 μg/cm^2^) and in vivo (12.5 - 100 μg) are also higher than those used by other investigators. For example, Wang et al. [12] and Azad et al. [38] used SWCNT with doses of 0.02 - 0.2 μg/cm^2^ in vitro and 10 μg/mouse in vivo, and 5-25 μg/ml in vitro, respectively. However, the mass of an individual CNT differs according to structure (SW vs MW). NM400 and NM402 are composed of 10-20 and 6-15 walls, respectively, leading to a mass per nanotube about 10-fold greater compared to SWCNT, for similar lengths. The rationale for this comparison implies that nanotube number is the relevant dose metrics accounting for the biological activity of CNT, which is a plausible hypothesis [3]. However, a study by Wang et al. [14] also showed the stimulation of fibroblast proliferation with a MWCNT concentration as low as 5 μg/ml in vitro. Differences in concentrations may also be related to different experimental procedures, such as cell lines, incubation time periods (48 h instead of 24 h) and presence of 10% serum during exposure to CNT (no serum in the present study).

Several authors have reported that the dispersion of CNT is a key determinant of their capacity to stimulate the proliferation of fibroblasts or to induce an interstitial fibrotic reaction in vivo [13,14,16]. Initially, we dispersed CNT in an aqueous solution containing FBS. However, we observed that serum had an additive effect on CNT activity on fibroblast proliferation, suggesting that serum growth factors could adsorb on CNT and be released during cell exposure. Indeed, we showed that the higher the FBS concentration used for dispersion, the stronger the proliferative effect on fibroblasts. It is important to notice that final serum concentrations (0.2% FBS) during exposure to CNT was identical whatever the initial dispersion condition. To avoid the effect of serum, we used a dispersion medium containing BSA, a protein that most likely adsorb onto CNT but has no effect on fibroblast survival or proliferation. Our choice is in agreement with previous studies recommending the use of BSA as a dispersant [14]. Furthermore, the final BSA concentration in exposure medium is lower than the concentration measured in the alveolar fluid of a normal mouse [33].

The pro-fibrotic activity of CNT in vitro was confirmed on different fibroblast cells of various origins, among which primary murine lung fibroblasts, mouse embryonic and lung fibroblast cell lines and human fetal lung fibroblasts, indicating that this activity is not species-specific and providing some support to extrapolate mouse in vivo and in vitro data to humans (parallelogram approach, [39]).

As first reported by Worle-Knirsch et al. [21], some nanospecific (CNT) properties can lead to interferences between NM and commonly used in vitro assays. Therefore, it is strongly recommended to take possible interferences into account and to verify results with other assays. Here, three tests based on totally different readouts were performed in order to confirm the proliferative effect of MWCNT. The data obtained with cell counting and the WST-1 assay indicated a CNT-stimulating effect on fibroblast proliferation. Since a reduction of cell death or apoptosis of fibroblasts could also account for these results, we confirmed the stimulation of fibroblast proliferation by using PI that detects cells in the process of DNA replication or division. The possible interferences between NM and WST-1 absorption wavelength or NM reactivity with WST-1 substrate have been integrated in our protocol by using triton-treated blanks for each condition. Thus, we have optimized the WST-1 assay for NM and results were validated by two other assays. We conclude that stimulation of fibroblast proliferation can be consistently assessed with the NM-adapted WST-1 assay described in this manuscript.

Finally, we validated our in vitro test by relating the results obtained with CNT, asbestos and silica to the development of pulmonary fibrosis in mice. NM400 and NM402 MWCNT and crocidolite induced fibrotic responses. NM400, however, had a stronger effect than crocidolite (asbestos), strengthening the concern about the hazardous properties of CNT. As CNT length is suggested to play an important role in CNT toxicity and particularly the pro-fibrotic potential [30,40] shortened NM400 (NM400c) and MWCNTg 2400 were tested. Interestingly, we observed that nanotube length/structure was critical since these shorter MWCNT did not induce fibroblast proliferation or lung fibrosis in mice contrary to NM400 and NM402. In contrast, in a previous study from our group [24], MWCNTg 2400 were shown to induce lung fibrosis. This apparent discrepancy could be explained by the different protocol conditions of the two studies: animal model (mouse vs rat), dispersion method (BSA + sonication vs Tween), mode of particle administration (aspiration injection vs intratracheal aspiration), dose (100 μg/mouse vs 2 mg/rat). Our results also indicate that metallic contaminants are not necessary for CNT pro-fibrotic activity since NM400c, chemically identical to NM400, was inactive in vitro and in vivo. Furthermore, we observed that NM400 and NM402 do not produce reactive oxygen species (ROS) in absence of cells [41], but instead exhibit a remarkable radical scavenging capacity, suggesting that acellular ROS production by CNT is also not necessary for CNT pro-fibrotic activity.

## Conclusions

The present study proposes an experimentally validated in vitro assay suitable for assessing the lung fibrogenic activity of NM. The results show that the in vitro proliferative activity of CNT strongly reflects the in vivo fibrosis findings, supporting a predictive value of our in vitro assay. The results obtained in this study also indicate that the structure/length of CNT constitute an important physicochemical determinant in the capacity of these materials to induce lung fibrosis. Several other CNT features have been shown to modulate their pro-fibrotic activity, reinforcing the need of a predictive test. Thus, the value of the present assay will need to be confirmed with a larger range of CNT covering different physicochemical properties and through an inter-laboratory comparison study to address its reproducibility and transferability. A better understanding of the cellular mechanism involved in the proliferative activity of CNT will further contribute to better establish the predictive value of the assay.

## Methods

### CNT, silica and asbestos

MWCNT NM400 and NM402 were obtained from the European Commission Joint Research Centre (Ispra, Italy) and originate from Nanocyl (Belgium) and Arkema (France), respectively. A fraction of NM400 was crushed in an oscillatory agate ball mill (Fritsch, Idar-Oberstein, Germany), with a vertical vibration of 1 mm applied during 6 h to obtain NM400c. MWCNTg 2400 were obtained as reported previously [24,25]. Briefly, these CNT were modified by grinding and then heated at 2400°C for the elimination of metal clusters and ablation of structural defects. Asbestos (crocidolite) was obtained from the Union internationale contre le Cancer (IUCC, Geneva, Switzerland) and Min-U-Sil (crystalline SiO_2_) from the U.S. Silica Company (Berkeley Springs, USA).

### Physicochemical characterization of MWCNT

Different analytical techniques were used to characterize MWCNT. Scanning electron microscopy (SEM) was used to study the length and diameter of the samples. CNT have been dispersed in a solution of chloroform (Sigma Aldrich, St Louis, USA) or in dispersion medium after sonication (see below) at room temperature and a drop of the fresh suspension was placed on a sample holder with a carbon coating and air dried. SEM pictures were taken with a scanning electron microscope 7600 F (Jeol, Tokyo, Japan) and NM400c length and diameter were measured with the Analysis program (Olympus, Tokyo, Japan). For NM400 and NM402, refer to [42] and for MWCNTg 2400, refer to [24,25]. Raman spectroscopy was acquired with a DXR SmartRaman Spectrometer (Thermo Scientific, Waltham, USA) operating in the microscopic mode with a 2048 pixel charge-coupled device (CCD) detector. A laser with an incident excitation wavelength of 780 nm and a power of 10 mW, a 50 μm slit aperture and a 10 times magnification objective were used to evaluate the degree of crystallinity of CNT. The concentration of metals in the samples was quantified after mineralization in acid (HNO_3_ 14 N and HCl 12 N; Merck, Darmstadt, Germany) in a high pressure microwave (Multiwave, Anton Paar GmbH, Graz, Austria). The amount of Fe was measured by atomic absorption spectrometry on a Spectra AA 300 (Varian Zeeman Inc., Palo Alto, USA) equipped with a graphite furnace atomizer and a Zeeman system for correction of nonspecific absorbance. The amount of Al and Co was measured by inductively coupled plasma mass spectrometry (ICP-MS; Agilent 7500G, Santa Clara, USA) with an octapole reaction system.

### Material dispersion

MWCNT were weighed in a sterile glass vial and suspended to a concentration of 2.55 mg/ml. Several dispersion media were evaluated. They were prepared with autoclaved nanopure H_2_O supplemented either with 2 or 10% fetal bovine serum (FBS, Invitrogen, Paisley, USA), 1.4 mg/ml bovine serum albumin (BSA, Sigma Aldrich, St Louis, USA) or 150 μg/ml Survanta (Abbott Laboratory, Illinois, USA). The suspension was sonicated (Virsonic 300 ultrasonic cell disrupter, Virtis, Gardiner, USA) for 16 min without interruption (power 15%) on ice. In order to keep the final concentration of the dispersant equal at all tested doses, the stock solutions (2.55 mg/ml) were serially diluted (2 fold-dilutions) in the same dispersion medium and then sonicated 15 min in a waterbath. Min-U-Sil and asbestos were directly dispersed with autoclaved nanopure H_2_O supplemented with 2% FBS or 1.4 mg/ml BSA.

### Fibroblast culture

Primary mouse lung fibroblasts were obtained from eight to twelve-week old female C57BL/6 mice (local breeding facility, Animalerie Centrale, Université catholique de Louvain, Brussels, Belgium), housed in positive pressure air-conditioned units (25°C, 50% relative humidity) on a 12 h light/dark cycle with free access to water and laboratory animal food. Mice were euthanized with an injection of pentobarbital (15 mg/mouse intraperitoneally, Certa, Braine-l'Alleud, Belgium). Whole lungs were perfused with phosphate buffered saline (PBS, Invitrogen) via the right ventricle. Lungs were minced with scissors for 4 min and then digested in 9 ml Hank's buffered salt solution (HBSS, Invitrogen), 0.8 ml Liberase TH (5 mg/ml, Roche, Mannheim, Germany) and 0.25 ml DNase (1 mg/ml, Worthington Biochemical Corporation, Lakewook, USA) at 37°C during 35 min under gentle agitation. The resulting cell suspension was filtered (70 μm), resuspended in Dulbecco’s Modified Eagle Medium (DMEM) supplemented with 10% FBS and 1% antibiotic-antimycotic (AA, Invitrogen), transferred into a flat tissue culture flask (2 lungs/75 cm^2^) and incubated at 37°C under 5% CO_2_. After 1 day, cells were washed with 10 ml DMEM and supplemented with fresh DMEM, 10% FBS, 1% AA. Cells were harvested 6 days later by adding 3 ml of 0.25% trypsin-EDTA (Invitrogen) and incubated for 10 min at 37°C. Trypsin was neutralized by adjunction of DMEM, 10% FBS and 1% AA. Cell suspensions were centrifuged for 10 min at 400 *g* and resuspended in 5 ml fresh DMEM, 10% FBS, 1% AA. Cell number and viability were determined with Trypan Blue Solution (Sigma Aldrich). Cells were seeded into 96-well or 24-well culture plates in complete medium (10% FBS) at a density of 30 000 or 180 000 cells/well, respectively, and incubated at 37°C under 5% CO_2_ for 24 h. Primary fibroblasts were sub-cultured for a maximum of 3 passages and used at passages 2 and 3 (refer to [43] for cell purity).

Mouse lung fibroblasts (MLg cell line) obtained from ATCC (catalog n°CCL-206) were cultured in MEM (Invitrogen) supplemented with 10% FBS and 1% AA and maintained at 37°C under 5% CO_2_. Cells were harvested, counted and cultured as previously described for primary fibroblasts. MLg were sub-cultured for a maximum of 15 passages.

BALB-3T3 cells were obtained from Pr. Michiels (Institut De Duve, Université catholique de Louvain, Brussels, Belgium) and cultured in DMEM GlutaMAX (Invitrogen) supplemented with 10% FBS and 1% AA at 37°C under 5% CO_2_. Cells were harvested, counted and cultured as previously described for primary fibroblasts. BALB-3T3 were sub-cultured for a maximum of 15 passages.

Human fetal lung fibroblasts (HFL-1 cell line) cells obtained from ATCC (catalog n°CCL-153) were cultured in F12K Nutrient Mixture (Invitrogen) supplemented with 10% FBS and 1% AA and maintained at 37°C under 5% CO_2_. Cells were harvested, counted and cultured as previously described for primary fibroblasts. HFL-1 were sub-cultured for a maximum of 15 passages.

### Fibroblast exposure

After 24 h at 37°C in complete culture medium (10% FBS), cells were washed once with their basal medium (no FBS, no AA) and then supplied with 200 μl (96-well culture plate) or 1 ml (24-well culture plate) medium with 1% AA containing 1/10 material dilutions. Cells were exposed during 24 h to 1 to 37.5 μg MWCNT or equivalent/cm^2^. Exposure to human platelet-derived growth factor (PDGF-BB, R&D System, Minneapolis, USA) at the indicated final concentration was used as a positive control for proliferation assays.

### Proliferation assays

Cell proliferation was assessed by cell counting, WST-1 assay and propidium iodide staining.

For cell counting, cells exposed for 24 h to test materials in 96-well plates were washed twice with their basal medium and then incubated with 25 μl 0.25% trypsin-EDTA for 10-15 min at 37°C. Trypsin was neutralized by adjunction of 100 μl of complete medium and cell suspensions were transferred and kept on ice. Cell number and viability were determined with Trypan Blue Solution on a Bϋrker cell. Data are presented as relative cell count compared to the non-exposed cells.

WST-1 is a colorimetric assay that quantifies mitochondrial activity and reflects cell viability. For this test, cells exposed for 24 h to test materials in 96-well plates were washed twice with their basal medium. Half of the cultures were supplied with 100 μl of medium and the other half with 100 μl medium containing 0.2% Triton X-100 to kill cells. Triton-treated wells were used as blanks since they contain the same amount of test material as test samples but have no mitochondrial activity. This procedure was introduced to eliminate the possible interferences of test materials with the assay either by interacting with the absorbance wavelength and/or with the WST-1 substrate. After 15 min at 37°C, 100 μl WST-1 (diluted 10 times in medium, Roche Diagnostics GmbH, Mannheim, Germany) were added to the cells and absorbance was measured after 60–90 min at 480 nm and at 680 nm (Spectrophotometer Infinite F200, Tecan, Mannedorf, Switzerland). Data are presented as relative cell activity (Abs _exposed_ / Abs _non-exposed_) where Abs = (Abs _480_ – Abs _680_) _no Triton_ – (Abs _480_ – Abs _680_) _Triton_.

For the propidium iodide (PI) staining, cells exposed for 24 h to test materials in 24-well plates were washed once with 500 μl MEM and then incubated with 200 μl 0.25% trypsin-EDTA for 10–15 min at 37°C, until cells detached. Trypsin was neutralized by adding 800 μl MEM, 10% FBS, 1% AA. Cell suspensions were centrifuged for 5 min at 100 *g* (4°C), washed with 1 ml PBS and then re-centrifuged for 5 min at 100 *g* (4°C). Cells were re-suspended in 1 ml EtOH 70% for fixation, by adding first 300 μl PBS and then 700 μl cold EtOH 100% drop wise while gently vortexing to avoid precipitation. Cells were incubated for at least 2 h at 4°C. Cell suspensions were then centrifuged for 5 min at 100 *g* (4°C), washed with 1 ml PBS, and then re-centrifuged for 5 min at 100 *g* (4°C). Then cells were resuspended in 250 μl PI staining solution containing 50 μg/ml PI (P4170, Sigma-Aldrich), Triton X-100 0.1% and 0.1 mg/ml RNase A (R6513, Sigma Aldrich), to ensure that only DNA was stained. Cells were filtered (40 μm), incubated 15 min at 37°C and then stored protected from light at 4°C. The samples (10000 cells) were analyzed by flow cytometry with BD FACS CANTO II (BD Biosciences, San Jose, USA) and the software BD FACS Diva. Pulse processing was used to exclude cell doublets and interference from the analysis. A first gate was applied to the scatter plot FSC vs SSC to remove cell debris and a second gate to the scatter plot PI-A vs PI-W to remove cell doublets. Cell% in G_0_-G_1_, S and G_2_-M phases were then deduced from the histogram plot event count vs PI derived from gates applied.

### Animals and treatments

Eight week-old C57BL/6 female mice were obtained from Taconic Europe (Lille Skensvad, Denmark), housed in positive pressure air-conditioned units (25°C, 50% relative humidity) on a 12 h light/dark cycle with free access to water and laboratory animal food. MWCNT (NM400, NM402, NM400c, MWCNTg 2400), crocidolite and Min-U-Sil were dispersed as previously described. In the first in vivo experiment, NM400 and NM402 suspensions were diluted to 100, 50, 25, 12.5 μg/50 μl H_2_O with 2% FBS. In the second experiment, NM400, NM400c, MWCNTg 2400 and crocidolite suspensions were only diluted to 100 μg/50 μl H_2_O with 1.4 mg/ml BSA. After anesthesia with a mix of Ketalar, 1 mg/mouse (Warner-Lambert, Zaventem, Belgium), and Rompun, 0.2 mg/mouse (Bayer, Leverkusen, Germany) given intraperitoneally, 50 μl of particle suspensions were injected directly into the lungs by pharyngeal aspiration. At least five mice were included per group. Control animals were treated with an equivalent volume of sterile water with the dispersant (control group). Positive control groups were instilled with 2.5 mg Min-U-Sil particles. Mice were sacrificed 2 months after instillation with an overdose of sodium pentobarbital (15 mg/mouse intraperitoneally). The left lobe was isolated by clamping the corresponding bronchi. This lobe was recovered in 3.65% paraformaldehyde (Sigma-Aldrich) in PBS for later histological analysis. The remaining lobes were lavaged to recover bronchoalveolar lavages (BAL), perfused with NaCl 0.9%, excised and then placed in 3 ml ice-cold PBS for determination of lung hydroxyproline (OH-proline) content.

### BAL and biochemical assays

BAL were performed by cannulating the trachea and lavaging the lobes with 1 ml NaCl 0.9%. The lavage was centrifuged (200 *g*, 10 min, 4°C) and the cell-free supernatant (BALF) was used for biochemical measurements. Enzyme-linked immunosorbent assays (ELISA) were performed on BALF according to manufacturer’s instructions (R&D System) for active transforming growth factor (TGF)-β1, platelet transforming growth factors (PDGF)-BB and osteopontin (OPN).

### Lung homogenates and measurement of lung collagen content

Lungs were homogenized on ice with an Ultra-Turrax T25 homogenizer (Janke & Kunkel, Brussels, Belgium) and stored at −80°C. Lung total collagen was estimated by measuring OH-proline, a specific component of collagen. Part of lung homogenate was hydrolyzed in HCl 6 N at 108°C during 24 h and OH-proline was quantified by high-performance liquid chromatography [44].

### Histology

Paraffin-embedded sections were stained with Sirius Red for light microscopy examination. Sirius red binds to total collagen, whereas the fast green stains non-collagenous proteins.

### Statistical analysis

Data are presented as means ± standard error on the mean (SEM). Differences were evaluated by using t-test or one-way analysis of variance, followed by Dunnett's Multiple Comparison or Newman-Keuls Multiple Comparison tests, as appropriate. Statistical significance was considered at *p* ≤ 0.05. Data analysis was performed with GraphPad Prism 5.00 (GraphPad Software, San Diego, USA).

## Abbreviations

AA: Antibiotic-antimycotic; BAL: Bronchoalveolar lavage; BALF: Bronchoalveolar lavage fluid; BSA: Bovine serum albumin; CNT: Carbon nanotube; DMEM: Dulbecco’s modified eagle medium; ECM: Extracellular matrix; FACS: Fluorescence-activated cell sorting; FBS: Fetal bovine serum; HBSS: Hank's buffered salt solution; HFL-1: Human fetal lung fibroblast; ICP-MS: Inductively coupled plasma mass spectrometry; IL: Interleukin; IUCC: Union internationale contre le cancer; MLg: Mouse lung fibroblast; MWCNT: Multi-wall carbon nanotube; NM: Nanomaterial; OPN: Osteopontin; PBS: Phosphate buffered saline; PDGF: Human platelet-derived growth factor; PI: Propidium iodide; ROS: Reactive oxigen species; SEM: Scanning electron microscopy; SWCNT: Single-wall carbon nanotube; TGF: Transforming growth factor; WST: Water-soluble tetrazolium salts.

## Competing interests

The authors declare that they have no competing interests.

## Authors’ contributions

GV is research fellow with the Fonds National de la Recherche Scientifique (FNRS, Belgium) and contributed to the experimental design, carried out in vitro and in vivo experiments, analyzed the experimental results and drafted the manuscript. SI participated in in vitro studies and ELISA analysis; MP and YY participated in animal exposure studies; CB participated in particle characterization; IF contributed in the particle characterization; EM carried out lung histology; DL and SV conceived the study, contributed to the experimental design, the evaluation of results and writing of the manuscript. SV also participated in the experimental work. All authors read and approved the final manuscript.

## References

[B1] Gorman J Taming high-tech particles: cautious steps into the nanothech future Sci News 2002 161 200 201 10.2307/4013155

[B2] Sanchez VC Pietruska JR Miselis NR Hurt RH Kane AB Biopersistence and potential adverse health impacts of fibrous nanomaterials: what have we learned from asbestos? Wiley Interdiscip Rev Nanomed Nanobiotechnol 2009 1 511 529 10.1002/wnan.41 20049814 PMC2864601

[B3] Donaldson K Aitken R Tran L Stone V Duffin R Forrest G Alexander A Carbon nanotubes: a review of their properties in relation to pulmonary toxicology and workplace safety Toxicol Sci 2006 92 5 22 10.1093/toxsci/kfj130 16484287

[B4] Lam CW James JT McCluskey R Hunter RL Pulmonary toxicity of single-wall carbon nanotubes in mice 7 and 90 days after intratracheal instillation Toxicol Sci 2004 77 126 134 14514958 10.1093/toxsci/kfg243

[B5] Shvedova AA Kisin ER Mercer R Murray AR Johnson VJ Potapovich AI Tyurina YY Gorelik O Arepalli S Schwegler-Berry D Unusual inflammatory and fibrogenic pulmonary responses to single-walled carbon nanotubes in mice Am J Physiol Lung Cell Mol Physiol 2005 289 L698 L708 10.1152/ajplung.00084.2005 15951334

[B6] Muller J Huaux F Moreau N Misson P Heilier JF Delos M Arras M Fonseca A Nagy JB Lison D Respiratory toxicity of multi-wall carbon nanotubes Toxicol Appl Pharmacol 2005 207 221 231 10.1016/j.taap.2005.01.008 16129115

[B7] Shvedova AA Kisin E Murray AR Johnson VJ Gorelik O Arepalli S Hubbs AF Mercer RR Keohavong P Sussman N Inhalation vs. aspiration of single-walled carbon nanotubes in C57BL/6 mice: inflammation, fibrosis, oxidative stress, and mutagenesis Am J Physiol Lung Cell Mol Physiol 2008 295 L552 L565 10.1152/ajplung.90287.2008 18658273 PMC2575941

[B8] Pauluhn J Subchronic 13-week inhalation exposure of rats to multiwalled carbon nanotubes: toxic effects are determined by density of agglomerate structures, not fibrillar structures Toxicol Sci 2010 113 226 242 10.1093/toxsci/kfp247 19822600

[B9] Mitchell LA Gao J Wal RV Gigliotti A Burchiel SW McDonald JD Pulmonary and systemic immune response to inhaled multiwalled carbon nanotubes Toxicol Sci 2007 100 203 214 10.1093/toxsci/kfm196 17660506

[B10] Ma-Hock L Treumann S Strauss V Brill S Luizi F Mertler M Wiench K Gamer AO van Ravenzwaay B Landsiedel R Inhalation toxicity of multiwall carbon nanotubes in rats exposed for 3 months Toxicol Sci 2009 112 468 481 10.1093/toxsci/kfp146 19584127

[B11] Morimoto Y Hirohashi M Kobayashi N Ogami A Horie M Oyabu T Myojo T Hashiba M Mizuguchi Y Kambara T Pulmonary toxicity of well-dispersed single-wall carbon nanotubes after inhalation Nanotoxicology 2012 6 766 775 10.3109/17435390.2011.620719 21942532

[B12] Wang L Mercer RR Rojanasakul Y Qiu A Lu Y Scabilloni JF Wu N Castranova V Direct fibrogenic effects of dispersed single-walled carbon nanotubes on human lung fibroblasts J Toxicol Environ Health A 2010 73 410 422 10.1080/15287390903486550 20155582

[B13] Wang L Castranova V Mishra A Chen B Mercer RR Schwegler-Berry D Rojanasakul Y Dispersion of single-walled carbon nanotubes by a natural lung surfactant for pulmonary in vitro and in vivo toxicity studies Part Fibre Toxicol 2010 7 31 10.1186/1743-8977-7-31 20958985 PMC2970581

[B14] Wang X Xia T Ntim SA Ji Z George S Meng H Zhang H Castranova V Mitra S Nel AE Quantitative techniques for assessing and controlling the dispersion and biological effects of multiwalled carbon nanotubes in mammalian tissue culture cells ACS Nano 2010 4 7241 7252 10.1021/nn102112b 21067152 PMC3899393

[B15] Wang X Xia T Duch MC Ji Z Zhang H Li R Sun B Lin S Meng H Liao YP Pluronic F108 coating decreases the lung fibrosis potential of multiwall carbon nanotubes by reducing lysosomal injury Nano Lett 2012 12 3050 3061 10.1021/nl300895y 22546002 PMC4143198

[B16] Wang X Xia T Ntim SA Ji Z Lin S Meng H Chung CH George S Zhang H Wang M Dispersal state of multiwalled carbon nanotubes elicits profibrogenic cellular responses that correlate with fibrogenesis biomarkers and fibrosis in the murine lung ACS Nano 2011 5 9772 9787 10.1021/nn2033055 22047207 PMC4136431

[B17] Crouch E Pathobiology of pulmonary fibrosis Am J Physiol 1990 259 L159 L184 2221080 10.1152/ajplung.1990.259.4.L159

[B18] Thannickal VJ Toews GB White ES Lynch JP 3rd Martinez FJ Mechanisms of pulmonary fibrosis Annu Rev Med 2004 55 395 417 10.1146/annurev.med.55.091902.103810 14746528

[B19] Lu P Takai K Weaver VM Werb Z Extracellular matrix degradation and remodeling in development and disease Cold Spring Harb Perspect Biol 2011 3 10.1101/cshperspect.a005058 PMC3225943 21917992

[B20] Mercer RR Hubbs AF Scabilloni JF Wang L Battelli LA Schwegler-Berry D Castranova V Porter DW Distribution and persistence of pleural penetrations by multi-walled carbon nanotubes Part Fibre Toxicol 2010 7 28 10.1186/1743-8977-7-28 20920331 PMC2958975

[B21] Worle-Knirsch JM Pulskamp K Krug HF Oops they did it again! carbon nanotubes hoax scientists in viability assays Nano Lett 2006 6 1261 1268 10.1021/nl060177c 16771591

[B22] Casey A Herwog E Davoren M Lyng FM Byrne HJ Chambers G Spectroscopic analysis confirms the interactions between single walled carbon nanotubes and various dyes commonly used to assess cytotoxicity Carbon 2007 45 1425 1432 10.1016/j.carbon.2007.03.033

[B23] Monteiro-Riviere NA Inman AO Zhang LW Limitations and relative utility of screening assays to assess engineered nanoparticle toxicity in a human cell line Toxicol Appl Pharmacol 2009 234 222 235 10.1016/j.taap.2008.09.030 18983864

[B24] Muller J Huaux F Fonseca A Nagy JB Moreau N Delos M Raymundo-Pinero E Beguin F Kirsch-Volders M Fenoglio I Structural defects play a major role in the acute lung toxicity of multiwall carbon nanotubes: toxicological aspects Chem Res Toxicol 2008 21 1698 1705 10.1021/tx800101p 18636756

[B25] Fenoglio I Greco G Tomatis M Muller J Raymundo-Pinero E Beguin F Fonseca A Nagy JB Lison D Fubini B Structural defects play a major role in the acute lung toxicity of multiwall carbon nanotubes: physicochemical aspects Chem Res Toxicol 2008 21 1690 1697 10.1021/tx800100s 18636755

[B26] Fubini B Fenoglio I Tomatis M Turci F Effect of chemical composition and state of the surface on the toxic response to high aspect ratio nanomaterials Nanomedicine (Lond) 2011 6 899 920 10.2217/nnm.11.80 21793679

[B27] Castranova V Schulte PA Zumwalde RD Occupational nanosafety considerations for carbon nanotubes and carbon nanofibers Acc Chem Res 2012 46 642 649 23210709 10.1021/ar300004a PMC4690205

[B28] Cesta MF Ryman-Rasmussen JP Wallace DG Masinde T Hurlburt G Taylor AJ Bonner JC Bacterial lipopolysaccharide enhances PDGF signaling and pulmonary fibrosis in rats exposed to carbon nanotubes Am J Respir Cell Mol Biol 2010 43 142 151 10.1165/rcmb.2009-0113OC 19738159 PMC2937228

[B29] Li R Wang X Ji Z Sun B Zhang H Chang CH Lin S Meng H Liao YP Wang M Surface charge and cellular processing of covalently functionalized multiwall carbon nanotubes determine pulmonary toxicity ACS Nano 2013 7 2352 2368 10.1021/nn305567s 23414138 PMC4012619

[B30] Wang P Nie X Wang Y Li Y Ge C Zhang L Wang L Bai R Chen Z Zhao Y Chen C Multiwall carbon nanotubes mediate macrophage activation and promote pulmonary fibrosis through TGF-beta/smad signaling pathway Small 2013 doi: 10.1002/smll.201300607. [Epub ahead of print] 10.1002/smll.201300607 23650105

[B31] Zanello LP Zhao B Hu H Haddon RC Bone cell proliferation on carbon nanotubes Nano Lett 2006 6 562 567 10.1021/nl051861e 16522063

[B32] Bhattacharya M Wutticharoenmongkol-Thitiwongsawet P Hamamoto DT Lee D Cui T Prasad HS Ahmad M Bone formation on carbon nanotube composite J Biomed Mater Res A 2011 96 75 82 21105154 10.1002/jbm.a.32958

[B33] Porter DW Hubbs AF Mercer RR Wu N Wolfarth MG Sriram K Leonard S Battelli L Schwegler-Berry D Friend S Mouse pulmonary dose- and time course-responses induced by exposure to multi-walled carbon nanotubes Toxicology 2010 269 136 147 10.1016/j.tox.2009.10.017 19857541

[B34] Phalen RF Basic morphology and physiology of the respiratory tract 1984 Boca Raton: Inhalation Studies: Foundations and Techniques CRC Press 33 75

[B35] Han JH Lee EJ Lee JH So KP Lee YH Bae GN Lee SB Ji JH Cho MH Yu IJ Monitoring multiwalled carbon nanotube exposure in carbon nanotube research facility Inhal Toxicol 2008 20 741 749 10.1080/08958370801942238 18569096

[B36] Galer DM Leung HW Sussman RG Trzos RJ Scientific and practical considerations for the development of occupational exposure limits (OELs) for chemical substances Regul Toxicol Pharmacol 1992 15 291 306 10.1016/0273-2300(92)90040-G 1509122

[B37] Donaldson K Murphy FA Duffin R Poland CA Asbestos, carbon nanotubes and the pleural mesothelium: a review of the hypothesis regarding the role of long fibre retention in the parietal pleura, inflammation and mesothelioma Part Fibre Toxicol 2010 7 5 10.1186/1743-8977-7-5 20307263 PMC2857820

[B38] Azad N Iyer AK Wang L Liu Y Lu Y Rojanasakul Y Reactive oxygen species-mediated p38 MAPK regulates carbon nanotube-induced fibrogenic and angiogenic responses Nanotoxicology 2013 7 157 168 10.3109/17435390.2011.647929 22263913 PMC4089498

[B39] Blaauboer BJ The applicability of in vitro-derived data in hazard identification and characterisation of chemicals Environ Toxicol Pharmacol 2002 11 213 225 10.1016/S1382-6689(01)00120-X 21782605

[B40] Poland CA Duffin R Kinloch I Maynard A Wallace WA Seaton A Stone V Brown S Macnee W Donaldson K Carbon nanotubes introduced into the abdominal cavity of mice show asbestos-like pathogenicity in a pilot study Nat Nanotechnol 2008 3 423 428 10.1038/nnano.2008.111 18654567

[B41] Ghiazza M Vietti G Fenoglio I Njuguna J, Pielichowski K, Zhu H Carbon nanotubes (CNTs): properties, applications and toxicity Health and environmental risks of nanomaterials UK: Woodhead Publishing in press

[B42] Kermanizadeh A Pojana G Gaiser BK Birkedal R Bilanicova D Wallin H Jensen KA Sellergren B Hutchison GR Marcomini A Stone V In vitro assessment of engineered nanomaterials using a hepatocyte cell line: cytotoxicity, pro-inflammatory cytokines and functional markers Nanotoxicology 2013 7 301 313 10.3109/17435390.2011.653416 22263564

[B43] Huaux F Noel S Dhooghe B Panin N Lo Re S Lison D Wallemacq P Marbaix E Scholte BJ Lebecque P Leal T Dysregulated proinflammatory and fibrogenic phenotype of fibroblasts in cystic fibrosis PLoS One 2013 8 e64341 10.1371/journal.pone.0064341 23734196 PMC3667188

[B44] Biondi PA Chiesa LM Storelli MR Renon P A new procedure for the specific high-performance liquid chromatographic determination of hydroxyproline J Chromatogr Sci 1997 35 509 512 10.1093/chromsci/35.11.509 9358626

